# Germline Risk Contribution to Genomic Instability in Multiple Myeloma

**DOI:** 10.3389/fgene.2019.00424

**Published:** 2019-05-08

**Authors:** Siegfried Janz, Fenghuang Zhan, Fumou Sun, Yan Cheng, Michael Pisano, Ye Yang, Hartmut Goldschmidt, Parameswaran Hari

**Affiliations:** ^1^Division of Hematology and Oncology, Medical College of Wisconsin, Milwaukee, WI, United States; ^2^Department of Internal Medicine, The University of Iowa Roy J. and Lucille A. Carver College of Medicine, Iowa City, IA, United States; ^3^Holden Comprehensive Cancer Center, The University of Iowa Roy J. and Lucille A. Carver College of Medicine, Iowa City, IA, United States; ^4^Interdisciplinary Graduate Program in Immunology, The University of Iowa Roy J. and Lucille A. Carver College of Medicine, Iowa City, IA, United States; ^5^The Third Affiliated Hospital, Nanjing University of Chinese Medicine, Nanjing, China; ^6^Ministry of Education’s Key Laboratory of Acupuncture and Medicine Research, Nanjing University of Chinese Medicine, Nanjing, China; ^7^Medizinische Klinik V, Universitätsklinikum Heidelberg, Heidelberg, Germany; ^8^Nationales Centrum für Tumorerkrankungen, Heidelberg, Germany

**Keywords:** plasma cell malignancy, genetic predisposition, DNA damage response, DNA repair, cancer predisposition syndromes

## Abstract

Genomic instability, a well-established hallmark of human cancer, is also a driving force in the natural history of multiple myeloma (MM) – a difficult to treat and in most cases fatal neoplasm of immunoglobulin producing plasma cells that reside in the hematopoietic bone marrow. Long recognized manifestations of genomic instability in myeloma at the cytogenetic level include abnormal chromosome numbers (aneuploidy) caused by trisomy of odd-numbered chromosomes; recurrent oncogene-activating chromosomal translocations that involve immunoglobulin loci; and large-scale amplifications, inversions, and insertions/deletions (indels) of genetic material. Catastrophic genetic rearrangements that either shatter and illegitimately reassemble a single chromosome (chromotripsis) or lead to disordered segmental rearrangements of multiple chromosomes (chromoplexy) also occur. Genomic instability at the nucleotide level results in base substitution mutations and small indels that affect both the coding and non-coding genome. Sometimes this generates a distinctive signature of somatic mutations that can be attributed to defects in DNA repair pathways, the DNA damage response (DDR) or aberrant activity of mutator genes including members of the *APOBEC* family. In addition to myeloma development and progression, genomic instability promotes acquisition of drug resistance in patients with myeloma. Here we review recent findings on the genetic predisposition to myeloma, including newly identified candidate genes suggesting linkage of germline risk and compromised genomic stability control. The role of ethnic and familial risk factors for myeloma is highlighted. We address current research gaps that concern the lack of studies on the mechanism by which germline risk alleles promote genomic instability in myeloma, including the open question whether genetic modifiers of myeloma development act in tumor cells, the tumor microenvironment (TME), or in both. We conclude with a brief proposition for future research directions, which concentrate on the biological function of myeloma risk and genetic instability alleles, the potential links between the germline genome and somatic changes in myeloma, and the need to elucidate genetic modifiers in the TME.

## Genomic Instability in Myeloma

Loss of genomic stability control leading to large-scale chromosomal aberrations is a widely recognized hallmark of human cancer (Hanahan and Weinberg, 2000) including the hematopoietic malignancy, plasma cell myeloma a.k.a. multiple myeloma (MM). Aberrations of this sort include deletions, insertions, inversions and translocations that can be readily detected using conventional Giemsa banding or spectral karyotyping in tumor cells in metaphase of the mitotic cycle (Liyanage et al., 1996; Schröck et al., 1996). Fluorescence *in situ* hybridization (FISH) and other molecular cytogenetic methods can be used for interphase cells. Myeloma is a rare, difficult-to-treat and, in the majority of cases, incurable neoplasm of terminally differentiated, immunoglobulin-producing B lymphocytes called plasma cells that reside in the bone marrow. Just as it does in other blood and solid cancers, loss of genomic integrity also results in small-scale aberrations of the myeloma genome. These can be discerned with the assistance of next generation sequencing (NGS) of genomic DNA, including whole-exome sequencing (WES) and whole-genome sequencing (WGS).

NGS technology – a collection of new methods for DNA sequencing developed in the mid to late 1990s and implemented in commercial DNA sequencers by the turn of the millennium – has tremendously empowered researchers to assess genomic instability in myeloma, look for insights into myeloma development and progression, and consider new approaches to personalized myeloma treatment. In contrast to first-generation technology including Sanger sequencing, NGS technology is cost effective and highly scalable, which allows large portions of the genome, such as the protein-encoding exome (WES), or the entire genome (WGS), to be sequenced at once. High-throughput NGS methods include pyrosequencing, ion semiconductor/torrent sequencing, sequencing by synthesis or ligation, nanopore sequencing, and combinatorial probe anchor synthesis. Regardless which method will be chosen for a given project, strong biocomputational support and a stringent data analysis pipeline are required to produce reliable results (Bacher et al., 2018).

Small-scale aberrations include base substitution mutations (point mutations), small insertions and deletions (indels), loss of heterozygosity, and copy number changes that affect individual genes or circumscribed chromosomal domains. Genomic instability in cancer including myeloma – often referred to as chromosomal instability or CIN– is of great clinical significance because it underpins clonal diversification and adaptation processes that facilitate, to name two outcomes, increased tumor heterogeneity in the course of tumor progression and acquired drug resistance in response to therapy. Therefore, CIN determines, in part, the duration and depth of the treatment response in patients with myeloma, which impacts progression-free and overall survival of. This relationship is reflected in survival-associated CIN signatures in myeloma that may be used for prognostication purposes (Chung et al., 2013; Zhou et al., 2013). Telomere length, another measure of genomic instability, is also associated with survival in myeloma (Hyatt et al., 2017).

From a comparative tumor biology point-of-view, CIN is a long-recognized and prominent feature of plasma cell tumors (PCTs) that arise in mouse models of human myeloma and related disorders. This includes the classic model of inflammation-dependent peritoneal plasmacytoma in strain BALB/c mice (Janz et al., 1993; Muller et al., 1994; Liyanage et al., 1996; Coleman et al., 1997, 2000; Felix et al., 1999) developed by Dr. Michael Potter at the United States National Cancer Institute more than 50 years ago (Potter, 1962; Anderson and Potter, 1969). Also included are more recently designed, genetically engineered mouse models (GEMMs) of myeloma, e.g., one that is based on the loss of Rrm2b (ribonucleotide reductase regulatory TP53 inducible subunit M2B) (Chang et al., 2013), a key enzyme in *de novo* deoxyribonucleotide synthesis important for DNA damage repair. CIN is an active area of preclinical and clinical myeloma research that has not only unearthed an abundance of candidate myeloma progression genes (Zhou et al., 2013) but also holds promise for improved determination of the risk with which the myeloma precursor conditions, monoclonal gammopathy of undetermined significance (MGUS) and smoldering myeloma (SMM), transition to frank myeloma (Dutta et al., 2018a,b). Given the importance of the bone marrow microenvironment in the natural history of myeloma, it is worth noting that preliminary evidence indicates that genomic instability in myeloma may “spill over” to bystander cells in the tumor microenvironment (TME). One example of this, reviewed in greater depth elsewhere (Adamik et al., 2018), is the induction of genomic instability in bone marrow stromal cells (BMSCs) upon exposure to myeloma cells (Garayoa et al., 2009; Garcia-Gomez et al., 2014). An intriguing example of the opposite, i.e., induction of genomic instability in myeloma by cells in the TME, is the dendritic cell-mediated activation of AID (activation-induced cytidine deaminase) (Koduru et al., 2012).

**FIGURE 1:**
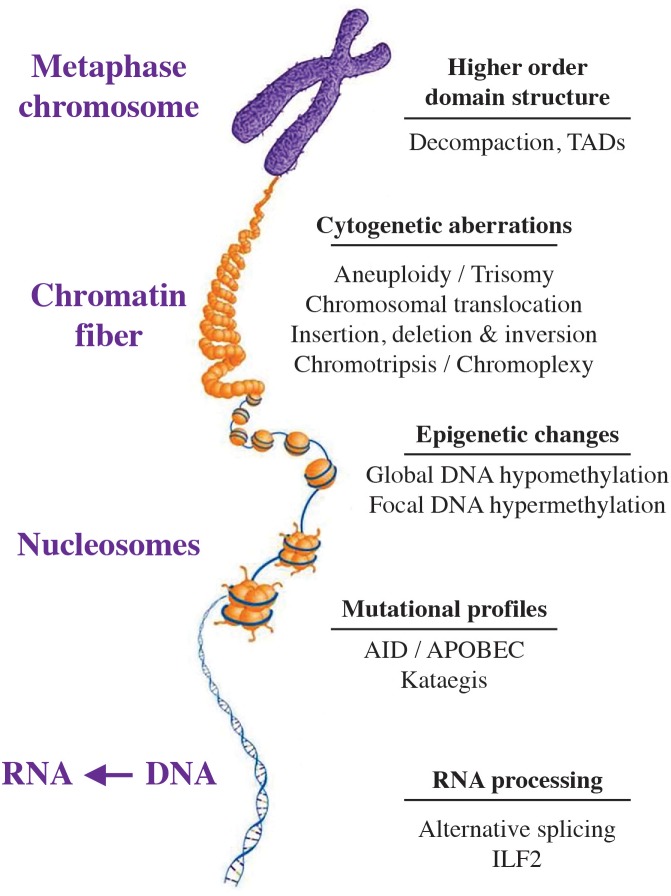
Manifestation of genetic instability at all levels of the myeloma genome. The hierarchical organization of the genome at the chromosomal, chromatin fiber, nucleosomal and nucleotide level is indicated by a scheme that is labeled. Genomic changes commonly seen in myeloma are listed on the right. Recent findings indicate that myeloma exhibits substantial epigenetic change that relies on a small set of transcription factors, including members of the IRF (interferon regulatory factor), ETS (E26 transformation-specific), MEF2 (myocyte-specific enhancer factor 2), E-Box (enhancer box) and AP-1 (activator protein 1) families of proteins. Also included are E proteins, such as TCF3 (transcription factor 3) a.k.a. E2A (E2A immunoglobulin enhancer-binding factors E12/E47), TCF4 (transcription factor 4) a.k.a. ITF-2 (immunoglobulin transcription factor 2), and TCF12 (transcription factor 12) (Jin et al., 2018). Jin et al. (2018) also showed that de-compaction of heterochromatin is a defining feature of myeloma cells, which is in line with evidence that the myeloma genome undergoes genome-wide DNA hypo-methylation in the course of tumor progression (Agirre et al., 2015). AID, activation-induced cytosine deaminase; APOBEC, apolipoprotein B mRNA editing enzyme, catalytic polypeptide; ILF2, interleukin enhancer binding factor 2; TADs, topologically associated domains.

Figure 1 shows that CIN manifests itself at all levels of the myeloma genome, spanning the chromosome and higher-order nuclear structure to individual genes. Presented in the section below is a short summary of forms, phenotypes and biological outcomes of genomic instability in myeloma, followed by a brief discussion of underlying sources and biological pathways. Additional information is available in expert reviews on genomic instability in cancer (Sansregret et al., 2018) including myeloma (Robiou du Pont et al., 2017) and high-risk myeloma (Pawlyn and Morgan, 2017).

### Cytogenetic and Mutational Landscape

From the cytogenetic perspective, recently reviewed by Kumar and Rajkumar (2018), MM can broadly be divided into neoplasms that harbor either a hyper-diploid genome due to trisomy that preferentially involves odd chromosomes or a pseudo- or hypo-diploid genome that contains a balanced (reciprocal) chromosomal translocation that recombines the immunoglobulin heavy-chain locus, *IGH*, at 14q32 with an oncogene on one of several partner chromosomes (Kuehl and Bergsagel, 2012; Morgan et al., 2012) – mainly with *MMSET* at 4p16 (Chesi et al., 1997, 1998b) or, less frequently, *MAF*, (Chesi et al., 1998a; Hurt et al., 2004) *MAFB* (Hanamura et al., 2001), *CCND1* (Bergsagel et al., 2005), and *CCND3* (Shaughnessy et al., 2001) at 16q23, 20q12, 11q13, and 6p21, respectively (Bergsagel and Chesi, 2013). That translocation-bearing myeloma karyotypes can be notoriously complex, presenting with the kind of “cytogenetic chaos” that is typically seen in solid but not hematopoietic cancers, has been recognized early on by cytogeneticists (Stewart and Fonseca, 2007). Chromothripsis and chromoplexy are recently discovered, extreme forms of chromosomal breakage and reassembly in myeloma cells that prognosticate poor survival (Kaur et al., 2018; Smetana et al., 2018). In keeping with the maxim that little if anything in myeloma is fully consistent, tumors carrying the Cyclin D1-activating t(11;14) translocation (≤20%) tend to have simple karyotypes (Robiou du Pont et al., 2017). What is more, approximately 10% of tumors exhibit no abnormality at all at the cytogenetic level (Avet-Loiseau et al., 2009). Myeloma cells also harbor recurrent unbalanced aberrations, most commonly gains at 1q and losses at 1p, 6q, 8p, 13q, 14q, 16q, and 17p (Carrasco et al., 2006; Avet-Loiseau et al., 2009). Gains and losses in these regions are thought to point, respectively, to putative myeloma onco- and suppressor genes – yet the nature of many of these genes remains obscure at this juncture. A newly identified cytogenetic subgroup of myeloma associated with a highly adverse risk profile features a hyper-haploid karyotype that contains but 30–33 chromosomes (Sawyer and Morgan, 2017; Sawyer et al., 2017). This subgroup is typically seen in younger patients and characterized by both multiple monosomies and loss of p53 function – the latter consequent to monosomy 17 and frequent mutations of *TP53* (Ashby et al., 2019; Peterson et al., 2019).

At the level of individual genes, myeloma exhibits a heterogenous, moderately affected mutational landscape that features a median of 60 somatic mutations detected by WES. In-depth analysis of WES results demonstrated that myeloma cells harbor a number of recurrently mutated genes but lack a consistent hallmark mutation such as the gain-of-function *MYD88*^L265P^ allele in Waldenström macroglobulinemia (Treon et al., 2012). The most commonly mutated genes in myeloma are *KRAS* and *NRAS* (∼20% of patients in both cases), followed by *TP53*, *DIS3*, *FAM46C*, and *BRAF* (∼10% in all cases) (Chapman et al., 2011). Additional mutations affecting *TRAF3, EGR1, SP140, FAT3* and a few other genes have been detected, but they are rare and not observed in more than ∼5% of patients (Walker et al., 2015a). Although limited to the exome (2% of the whole genome), the mutational analysis of primary tumor samples has yielded a better understanding of the clonal evolution of myeloma, including difficult questions such as whether mutations that target the same pathway (e.g., *KRAS-*, *NRAS-*, or *BRAF-*dependent activation of MAPK signaling) occur in the same cell clone or are distributed among different cell clones admixed in the same diagnostic bone marrow sample (Bolli et al., 2014). The two possibilities are difficult to distinguish by DNA sequencing. Panel sequencing of the genes mentioned above, which may soon arrive as a commercial assay in the clinic (Kortuem et al., 2016), will likely facilitate the selection of molecularly targeted drugs, an important step toward individualized myeloma treatment. Panel sequencing may also facilitate the detection of circulating myeloma cells in the peripheral blood (Lohr et al., 2016), a promising method that currently relies on genome-wide sequencing of cell-free DNA (cfDNA) (Guo et al., 2018; Waldschmidt et al., 2018). Panel sequencing can also be employed as discovery tool. For example, its clever use recently led to the surprising finding that myeloma cells may harbor kinase-activating fusion genes (Cleynen et al., 2017; Morgan et al., 2018) analogous to the *BCR-ABL1* fusion seen in t(9;22)^+^ chronic myeloid leukemia (CML).

### Mutational Targets, Drivers, and Signatures

Whole-genome sequencing (WGS) provides deep insight into the mutational landscape of myeloma because it covers the vast non-coding portion of the genome (98%) in addition to the protein-encoding portion (2%). WGS revealed that the myeloma genome is littered with many mutations (5–10 × 10^3^) in both transcribed and non-transcribed regions, with the former including many mutations that target microRNA, small nucleolar RNA and long-non-coding RNA amongst other RNA species (Morgan et al., 2014). The overwhelming majority of mutations detected by WGS are postulated to represent bystander or passenger mutations, i.e., “genetic noise” or “collateral damage” that results from compromised genomic integrity but is not relevant for tumor development and progression. Distinguishing mutational driver from bystander events is a major challenge going forward. A case in point are previously identified “driver” mutations in transcribed genes that were later on found to be barely expressed at the mRNA level (Rashid et al., 2014), which casts serious doubt upon the postulated tumor-promoting role of the mutations.

By virtue of uncovering distinct mutational signatures in gDNA, WGS has also made a major contribution to the identification of the genotoxic stress that underpins the mutational landscape of myeloma. Four signatures have been identified thus far: (1) methylated cytosine deamination, a generic mutational process observed in many cancers that results in cytosine-to-thymine (C**→**T) transitions at CpG (guanine) dinucleotide sites; (2) kataegis, a pattern of localized hypermutation that co-localizes with regions of genomic rearrangements and also leads to C**→**T transitions but in the context of TpC dinucleotides; (3) APOBEC (apolipoprotein B mRNA editing enzyme, catalytic polypeptide), a pathway of somatic mutagenesis that is most frequently found in tumors that harbor MAF or MAFB activating chromosomal translocations and targets C to undergo transition to T or transversion to G or A (adenine) at TpC sites; (4) AID (activation-induced cytosine deaminase), a mutator enzyme that is essential for V(D)J hypermutation and Ig isotype switching in normal B lymphocytes (Muramatsu et al., 2000), mechanistically involved in MYC-activating translocations in aberrant B cells (Ramiro et al., 2004), and able to mutagenize oncogenes in myeloma (e.g., *CCND1*) that are rearranged by illegitimate trans-chromosomal exchange with the *IGH* locus [e.g., t(11;14) translocation] (Walker et al., 2015b). The APOBEC signature is of particular interest due to its prognostic impact in myeloma (Maura et al., 2018).

A recent large-scale WGS analysis of newly diagnosed myeloma (NDMM) by Walker et al. (2018b) – supplemented with RNA-seq data and associated with the clinical and outcome results in nearly 1,300 patients – greatly expanded the list of putative myeloma oncogenes (*PTPN11, PRKD2, SF3B1, IDH1, and IDH2*) and tumor suppressor genes (*UBR5, HUWE1*). Interestingly, amongst a total of 63 driver genes, 17 are potentially actionable in terms of pharmacological targeting. Additionally, the WGS analysis shed light on myeloma progression pathways that exhibit tumor subtype-dependent preferences previously reported by Bolli et al. (2018a). One common emerging theme is transcription-coupled mutagenesis, i.e., mutations in oncogenes occur solely, or at an increased rate, in tumors in which the expression of these genes is constitutively upregulated by chromosomal translocation. Examples include elevated mutation frequencies in *CCND1* in t(11;14)^+^ tumors, as mentioned above; *MAF* in t(14;16)^+^ tumors; and *FGFR3* in t(4;14)^+^ myelomas. As pointed out by Bergsagel and Kuehl (2018), the mechanistic basis of other associations revealed by WGS analysis is less clear, e.g., prevalence of gains in 11q, mutations in *FAM46C* and rearrangements of *MYC* in hyper-diploid tumors. The preferred occurrence of *PRDK2* and *DIS3* mutations in t(4;14)^+^ tumors and the association of *BRAF*, *DIS3* and *ATM* mutations in t(14;20)^+^ tumors also lacks a mechanistic explanation at this time.

The study summarized above and earlier work by Bolli et al. (2018b) have redefined our understanding of genetic drivers of myeloma to include not only mutated driver genes but also chromosome gains and losses, chromosomal translocations, loss of heterozygosity, and the APOBEC mutational signature mechanism (Maura et al., 2018). The p53 tumor suppressor, encoded by *TP53*, is an example of a mutated driver that strongly predicts poor outcome. The short survival of patients with “double-hit” NDMM involving p53 (Walker et al., 2018a) and the prognostic value of sub-clonal p53 copy numbers (Shah et al., 2018) underline the clinical relevance of p53 as a target of and contributor to genomic instability in myeloma. Preliminary findings suggest that another tumor suppressor gene, *WWOX*, which is frequently involved in chromosomal translocation (Handa et al., 2017; Hussain et al., 2018), also falls into the category of driver genes that are able to amplify genomic instability once they have been targeted by somatic mutation.

### Deregulated DNA Damage Response

**FIGURE 2:**
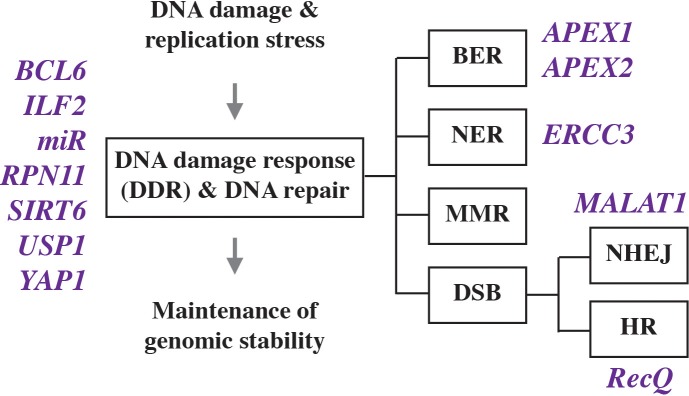
Genes involved in deregulated DNA damage response and defective DNA repair in myeloma.

All cells including myeloma are able to deal with a moderate level of genomic damage by activating a network of adaptive changes and biological pathways collectively termed DNA damage response (DDR). The response includes DNA damage recognition, checkpoint control, cell cycle arrest and, importantly, DNA repair. Depending on biological context, DDR leads to different outcomes, e.g., programmed cell death (apoptosis), which may be followed by immune clearance of apoptotic debris; senescence, which may support a state of tumor dormancy; and survival, the precondition for tumor precursors to continue on the path of neoplastic development and complete malignant transformation. The molecular events that comprise the DDR in mammalian cells have been elucidated in detail and grouped into three functional steps: “sensors” that recognize damage, “transducers” that coordinate and effect signal transduction, and “effectors” that execute biological outcomes. DNA repair is comprised of a variety of lesion-specific pathways that include mismatch repair (MMR), base excision repair (BER), nucleotide excision repair (NER), transcription coupled repair (TCR), and DNA double strand break (DSB) repair. The latter employs different molecular machineries and sub-pathways known as homologous recombination (HR), non-homologous end joining (NHEJ), microhomology mediated end joining (MMEJ), and Fanconi anemia (FA) repair. As recently reviewed by others (Herrero and Gutierrez, 2017), it is abundantly clear that myeloma cells manifest activated, dysfunctional DDR and DNA repair activities (Figure 2) that are involved in tumor development and also important for acquisition of resistance to myeloma drugs and patient survival.

**FIGURE 3:**
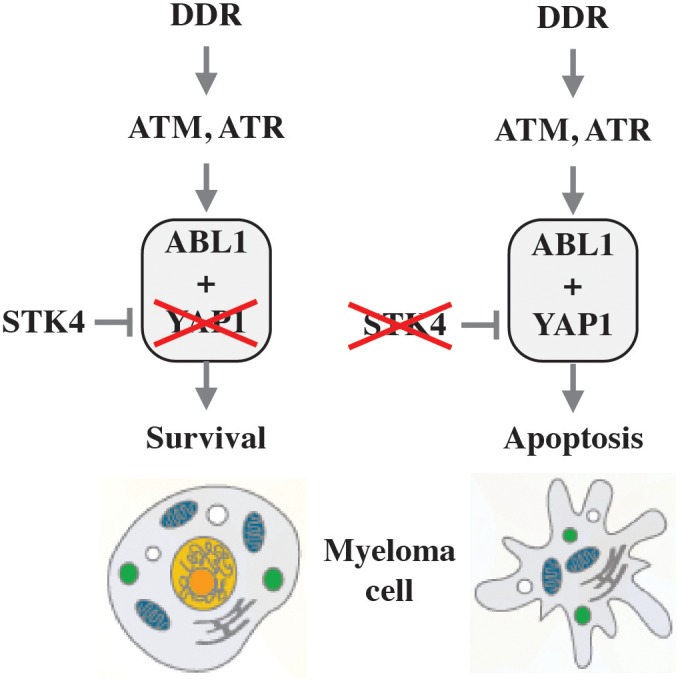
Killing myeloma by activating YAP1. Unlike normal cells, in which nuclear ABL1 triggers cell death via interaction with YAP1, this pathway is defect in myeloma due to low levels of YAP (indicated by red X in left panel). Since YAP1 is down regulated in myeloma cells by STK4, pharmacological inactivation of the kinase (denoted by red X in right panel) may restore YAP1 levels to the point at which programmed cell death is triggered.

While inactivation of p53 and loss of ATM or ATR function upstream of p53 are crucial oncogenic events in the natural history of solid tumors, changes of this sort are infrequent in myeloma and thus unlikely to govern the DDR in neoplastic plasma cells. On this backdrop, it is of great significance that Cottini et al. recently implicated YAP1 (Yes associated protein 1) in DNA damage-dependent apoptosis in myeloma (Cottini et al., 2014a,b). YAP1 is an activator of the Hippo signaling pathway that controls organ size by virtue of regulating cell proliferation and apoptosis and causes a hippopotamus-like phenotype of tissue overgrowth if hyperactivated by certain mutations. Cottini et al. showed that pervasive DNA damage in myeloma cells leads to activation of a p53-independent pro-apoptotic network that is centered on the nuclear re-localization of ABL1 kinase, which is widely known for its key role in CML and Philadelphia chromosome-positive (Ph^+^) acute lymphoblastic leukemia (ALL) and the development of the first-in-class molecularly targeted drug, imatinib (Gleevec^®^). Although nuclear ABL1 triggers cell death via interaction with YAP1 in normal cells, low YAP1 levels in myeloma – due to genetic inactivation or reduced expression – prevent nuclear ABL1-induced apoptosis (Figure 3, left). This may be relevant for myeloma treatment, because YAP1 is under control of the serine-threonine kinase, STK4, and pharmacological inactivation of STK4 may restore YAP1 levels and, thereby, kill myeloma cells (Figure 3, right). This provides the rational for the development of YAP1 activators (Maruyama et al., 2018) for patients with myeloma harboring low YAP1 levels. Of interest from the tumor development point-of-view, the study led to the intriguing hypothesis that inactivation of the ABL1-YAP1 axis may substitute for loss of p53 function in myelomagenesis.

The successful development of bortezomib (Velcade) and related next-generation inhibitors, now commonly used as backbone drugs for myeloma treatment, has moved the proteasome to the center stage of myeloma research. Recent findings have linked the regulation of protein homeostasis via ubiquitination and deubiquitination upstream of the proteasome with the DDR in myeloma. Ubiquitination is a sequential enzymatic process that covalently attaches the 76-residue polypeptide ubiquitin to client proteins, which targets them for proteasomal degradation or regulates functional properties such as enzymatic activity, subcellular localization and interaction with other proteins. Just like other post-translational modifications, ubiquitination can be reversed by a sizeable family of (*n* ≤ 100) deubiquitinases (DUBs). These can be classified into six subfamilies based on sequence and domain conservation, and are able to cleave ubiquitin from target proteins, edit ubiquitin chains on proteins, or process ubiquitin precursors in order to maintain a pool of free ubiquitin necessary for normal cell function (Harrigan et al., 2018). Das et al. (2017) recently demonstrated the involvement of the ubiquitin specific peptidase 1 (USP1) in the myeloma DDR, and showed that a small-drug USP1 inhibitor, designated SJB3-019A, decreases the viability of myeloma cells and overcomes bortezomib resistance (Figure 2, left). This relied on a mechanism that included the co-inhibition of the Fanconi anemia complex and the HR sub-pathway of DSB repair (Das et al., 2017). Similar findings were obtained in studies on another DUB known as proteasome regulatory particle lid subunit RPN11 (Song et al., 2017), for which a candidate small-molecule inhibitor, capzimin, is available as lead compound for further development (Li J. et al., 2017).

**FIGURE 4:**
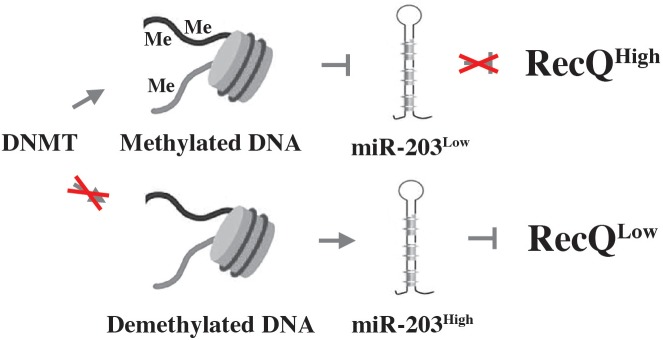
DNMT inhibition chemo-sensitizes myeloma using a mechanism that involves the down regulation of RecQ. Aberrant methylation-dependent repression of miR-203 leads to upregulation of RecQ by diminishing the efficacy with which miR-203 inhibits the expression of the helicase (indicated by red X in upper panel). High levels of RecQ in myeloma cells promote resistance to replication-dependent DNA damage and myeloma drugs. Treatment of myeloma cells with DNMTi (red X in lower panel) results in de-repression of miR-203 and downregulation of RecQ, causing loss of resistance to replication stress and myeloma drugs (bottom).

Myeloma’s DDR is also regulated via epigenetic mechanisms, as recently shown by studies on the role of the histone deacetylase, SIRT6 (sirtuin 6), in genomic stability control. SIRT6 is a NAD^+^ dependent enzyme that is highly expressed in myeloma cells and associated with adverse prognosis. The mechanism by which SIRT6 operates in myeloma depends in part on the downregulation of the mitogen-activated protein kinase (MAPK) pathway. This involves both interaction of SIRT6 with the ETS transcription factor, ELK1, and activation of DNA repair pathways via checkpoint kinase 1 (CHEK1), a serine-threonine kinase that coordinates DNA damage and cell cycle checkpoint responses (Cea et al., 2016). Another regulator of genomic stability in myeloma is RecQ helicase, a DNA-unwinding enzyme identified as one of the most downregulated genes in a genome-wide expression screen of myeloma responding to DNA methyltransferase (DNMT) inhibition (DNMTi) (Viziteu et al., 2017). RecQ, encoded by *RECQ1*, is significantly overexpressed in myeloma compared to normal plasma cells, and increased *RECQ1* message is associated with poor prognosis in patients with myeloma. Genetic downregulation of *RECQ1* induced cell death (apoptosis) and DSBs in myeloma (Figure 2, right), while upregulation of *RECQ1* protected from melphalan and bortezomib cytotoxicity. Mechanistically, the pharmacologic downregulation of *RECQ1* using DNMTi relies on a microRNA called miR-203 (Figure 4).

The above-mentioned study by Song et al. (2017) revealed an interesting parallel to a therapeutic vulnerability of breast, ovarian and other solid tumors that are sensitive to PARP (poly ADP-ribose polymerase) inhibition because they lack functional BRCA1 or BRCA2 tumor suppressor protein. In myeloma RecQ interacts with PARP, raising the possibility DNMTi synergizes with PARPi to kill myeloma cells in which RecQ is expressed at high levels. However, this has not been demonstrated. Following up on the finding on miR-203, researchers have implicated additional miRs in genomic instability in myeloma. Examples include the discovery of a miR-29b-dependent pathway (Botta et al., 2018), the finding that miR-137 induces genomic instability in an aurora kinase A (AURKA)-dependent manner (Qin et al., 2017) and the observation that regulation of DNA ligase III in myeloma involves miR-22 (Caracciolo et al., 2018). No doubt, the list of miRs is poised to expand as the field moves forward and additional RNA species will be tested. A long non-coding RNA (lncRNA) dubbed MALAT1 has also come into play (Hu et al., 2018) and the master regulator of B-cell development, BCL6, has been shown to down regulate the DDR in myeloma (Tahara et al., 2017).

### Defective DNA Repair and RNA Processing

Similar to the CIN score mentioned above, Bernard Klein and his associates devised a DNA repair score that is predictive of progression-free and overall survival of patients with myeloma (Kassambara et al., 2014). The risk score is based on the expression of 22 genes that encode DNA repair proteins in myeloma, with 17 and 5 genes linked to poor and good outcome, respectively. The score’s robustness underlines the impact of aberrant DNA repair in myeloma. Findings that myeloma backbone drugs such as alkylating agents (melphalan) and proteasome inhibitors (bortezomib) affect the capacity of myeloma cells to maintain genomic stability (Gourzones-Dmitriev et al., 2013) quickly led to the postulate that enhanced understanding of mechanisms of DNA repair in myeloma will lead to new therapeutic approaches based on the concept of synthetic lethality. This arises when a combination of deficiencies in two genes (e.g., gene X and a DNA repair gene) causes cell death, whereas a deficiency in only one of the genes (gene X) does not. The first example of a molecularly targeted drug that successfully exploited the concept of synthetic lethality (first FDA approval in 2014) is the development of PARP inhibitors for the treatment of solid tumors deficient in *BRCA1* and *BRCA2* function. These tumor suppressor genes are important for the error-free HR pathway of DSB repair. Interestingly, Neri et al. (2011) showed that myeloma cells may be pharmacologically sensitized to PARP inhibition by bortezomib-induced “BRCAness,” in which bortezomib-dependent impairment of HR results in synthetic lethality in combination with PARP inhibition.

Continuing with studies on HR-dependent DSB repair, several independent groups demonstrated that dysfunctional, elevated HR underlies genomic instability and increases the burden of genetic change that leads to drug resistance and disease progression in myeloma (Shammas et al., 2009; Kumar et al., 2018). An interesting new development is the finding that the BER-associated apurinic/apyrimidinic (AP) nucleases, APEX1 and APEX2, contribute in important ways to the regulation of HR in myeloma (Kumar et al., 2018). Genetic and pharmacological inhibition of APEX1 and APEX2 inhibited HR activity in myeloma cells, using a mechanism that involved the ability of AP nucleases to regulate the expression of RAD51 recombinase. RAD51 depends in part on the *TP73*-encoded tumor protein p73, which is related to p53 and also considered a tumor suppressor although debates about its role in malignant development persist. Another recent advance is the implication of NER in CIN in myeloma. Szalat et al. showed that expression of the canonical NER gene *ERCC3* (excision repair cross-complementation group 3) significantly impacted the outcome in newly diagnosed MM patients treated with alkylating agents (Figure 2, right). The investigators also demonstrated that targeting xeroderma pigmentosum complementation group B (XPB), the DNA helicase encoded by *ERCC3*, led to NER inhibition, which in turn significantly increased sensitivity to alkylating agents (Szalat et al., 2018).

There is also some preliminary evidence for mismatch repair (MMR) deficiency in myeloma detected with the help of a high-resolution florescent method of microsatellite instability (MSI) analysis (Oda et al., 2005). Following up on earlier observations suggesting the MSI phenotype occurs in ∼20% of myelomas (Velangi et al., 2004) or as many as ∼50% of myelomas (Timuragaoglu et al., 2009), Miyashita et al. (2018) recently used the high-resolution florescent MSI assay to unequivocally demonstrate microsatellite instability in 2 of 20 (10%) patients with myeloma – one at the time of diagnosis and the other in the course of disease progression. Although it appears MMR deficiency is not frequent in myeloma, it may be still be worthwhile to identify patients of this sort because the experience with solid tumors, particularly colorectal carcinoma, showed that MSI can determine the response to cancer immunotherapy. One striking example is long-term remissions in a subset of patients with metastatic disease treated with immune checkpoint inhibitors (Ganesh et al., 2019).

Post-transcriptional RNA processing adds another layer of complexity to the maintenance of genomic stability in myeloma (Marchesini et al., 2017a). RNA processing includes the concerted modification of the splicing patterns of transcripts involved in DNA repair and maintenance of genomic stability in response to genotoxic stress (Colla et al., 2015). The alternative splicing program governed by the DDR relies on the proper regulation of the expression, localization and activity of RNA-binding proteins (RBPs) that serve as gatekeepers of genomic integrity (Pereira et al., 2017). Since the disruption of the regulatory interplay between RBPs and DDR may promote genomic instability and acquisition of drug resistance, the targeting of aberrant RBP function during the DDR is an active area of preclinical myeloma research aimed at developing new approaches to sensitize myeloma cells to DNA damaging agents. The potential to therapeutically target aberrant RBP activities in myeloma has been demonstrated by Marchesini et al. The investigators showed that genomically unstable and aggressive myelomas carrying the 1q21 amplification have acquired dependency on the 1q21 induced overexpression of the RBP ILF2 (interleukin enhancer binding factor 2) (Marchesini et al., 2017b). ILF2 functions as a key modulator of HR repair in myeloma. Mechanistically, high ILF2 expression drives resistance to genotoxic agents by modulating the translocation of YB1 (Y-box binding protein 1) from the cytoplasm to the nucleus where it interacts with a splicing factor that promotes mRNA splicing of transcripts involved in HR repair. These findings are consistent with clinical observations that “1q21 patients” benefit less from high-dose therapy than non-1q21 patients, and that nuclear expression of ILF2 is highly correlated with that of YB1 in 1q21 myeloma. The findings also agree with laboratory results showing that YB1 downregulation following DNA damage leads to γH2AX accumulation and caspase 3 activation in myeloma cells. Importantly, the work by Marchesini et al. suggests that ILF2 may serve as a good biomarker of aggressive myeloma, and that blocking the ILF2 signaling axis may enhance the efficacy of myeloma therapies that are based on DNA-damaging agents.

### Plasma Cell Leukemia

Plasma cell leukemia (PCL), the most aggressive and deadly plasma cell neoplasia, features higher levels of genomic instability than myeloma. Primary plasma cell leukemia (pPCL) is a rare malignancy that is diagnosed in patients without a previous history of myeloma (Mina et al., 2019) and operationally defined by presence of ≥20% clonal plasma cells in the peripheral blood and/or an absolute number of more than 2 million leukemic plasma cells per mL of peripheral blood (Gavriatopoulou et al., 2018). pPCL must be distinguished from secondary PCL (sPCL) that arises in some patients with myeloma – usually those with end-stage relapsed and/or refractory disease or with the extra-medullary plasma cell tumor, plasmacytoma, that progresses for reasons that are not clear to a generalized, leukemic pattern of tumor cell dissemination. Compared to myeloma, PCL is characterized not only by elevated genomic instability resulting in large numbers of cytogenetic and molecular genetic aberrations (Avet-Loiseau et al., 2012) but also by a higher prevalence of other adverse clinical and laboratory features (van de Donk et al., 2012; Mina et al., 2017). Unlike MM, pPCL exhibits a predominantly non-hyperdiploid karyotype (Avet-Loiseau et al., 2001). Additional features include an unusually high frequency of t(11;14) translocations (Tiedemann et al., 2008), illegitimate rearrangements of the *MYC* locus at 8q24 (Tiedemann et al., 2008) and deletions of 13q, 17p, 1p21 with or without amplification of 1q21 (Chang et al., 2009). pPCL and myeloma share a pattern of global hypomethylation of the tumor genome (Todoerti et al., 2018). Due in large measure to the rarity of the disease and the lack of dedicated preclinical model systems that lend themselves to mechanistic studies, the genetic and biological pathways that underlie genomic instability in PCL are not known.

## Genetic Pre-Disposition to Myeloma

**FIGURE 5:**
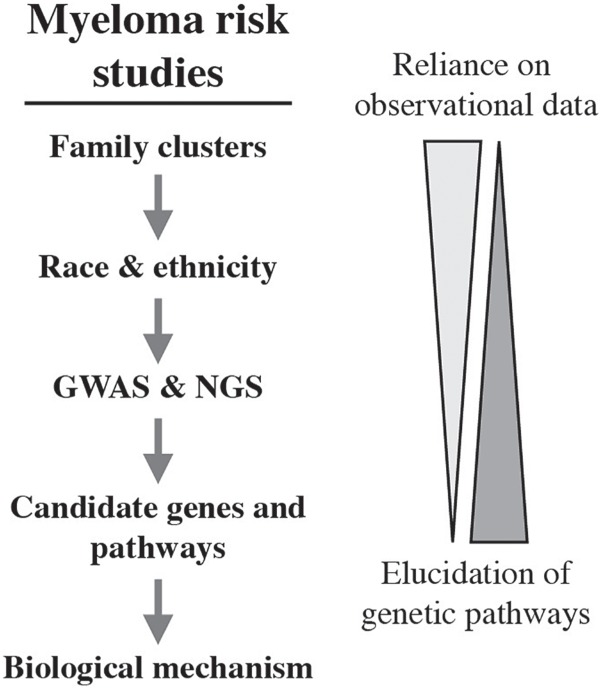
Observational, genomic and mechanistic studies in the discovery pipeline of inherited myeloma risk.

A comprehensive, insightful review on the inherited susceptibility to myeloma was published in 2014 (Morgan et al., 2014). It expertly covered epidemiological and genetic association studies on the risk of developing myeloma, including ethnic and racial differences, and discussed the association of myeloma risk alleles with cytogenetic and molecular subgroups of myeloma. The inherent challenges of defining myeloma risk at the individual level, e.g., a family member seeking counseling, are also considered. Five years later, hypothesis-free genetic association testing can be combined with newly available NGS technologies to deepen our understanding of the genetic networks that underpin the natural history of myeloma. An implicit promise of the new capabilities is uncovering novel molecular targets for the design and testing of innovative strategies to myeloma treatment and prevention. New biomarkers for the clinical management of myeloma, including individualized treatment plans, may also be uncovered. What follows below is a brief review of findings in myeloma risk research. This work began with the description of family clusters and recognition of racial disparities, continued with the identification of candidate risk loci, and is now putting a premium on annotating risk loci with biological functionality (Figure 5).

### Myeloma Risk Alleles Identified in Genome-Wide Association Studies

Genetic predisposition to myeloma was first discovered by clinical observation. Indeed, cases of familial myeloma with several family members affected by MGUS or MM have been reported since the 1920s. Epidemiological case control studies followed up on these initial findings. The largest study, published in 2010, analyzed 13,896 Swedish myeloma patients and 54,365 healthy controls. It revealed that first-degree relatives of people with myeloma experienced a higher relative risk (RR) for developing MM (RR 2.1) and MGUS (RR 2.1) but also acute lymphoblastic leukemia (RR 2.1) and, to a lesser extent, solid tumors (RR 1.1) (Kristinsson et al., 2009). This line of investigation was complemented by hypothesis-driven genetic association studies that utilized a candidate gene/pathway approach to identify genetic variants that influence myeloma risk. Analyses included polymorphisms in genetic networks one might intuitively implicate in the natural history of myeloma (e.g., cytokine-dependent immune response, DNA repair, and apoptosis) but also considered alternative hypotheses such as folate metabolism and insulin-like growth factor signaling. A number of positive associations with myeloma risk were reported, but none of these were independently replicated or free of potentially fatal flaws, such as insufficient sample size or cryptic relatedness of study probands (Morgan et al., 2014). These limitations were overcome by unbiased genome-wide association study, or GWAS.

GWAS is able to achieve the required stringent threshold of genome-wide significance (5 × 10^-8^) and can be readily set up to independently confirm candidate loci. Beginning in 2012, GWAS identified myeloma susceptibility loci on chromosomes 3p22, 7p15.3, 8q24, and 2p23.3 (Broderick et al., 2012; Martino et al., 2012), with allele frequencies (RAFs) and per-allele odds ratios (ORs) invariably indicating common and low-risk variants (Table 1). By 2016 additional GWAS and case-control studies had identified association signals for 17 risk variants (Chubb et al., 2013; Weinhold et al., 2013; Mitchell et al., 2016) – a number that has been increased to 23 in the most recent report (Went et al., 2018). Some risk variants, e.g., the one at16p13, are also associated with survival of patients with myeloma (Ziv et al., 2015).

**Table 1:** Myeloma risk variants discovered by GWAS analysis of patients with myeloma vs controls.

Chromosomal location^1^	RAF^2^	OR^3^	Gene or candidate gene^4^	Gene target^5^	Biological pathway^6^
2p23.3 (Broderick et al., 2012)	0.81	1.23	*DNMT3A*	DTNB	
2q31.1 (Went et al., 2018)	0.77	1.12	*SP3*	AICDA	Genomic instability
3p22.1 (Broderick et al., 2012)	0.16	1.26	*ULK4*		
3q26.2 (Chubb et al., 2013)	0.75	1.20	*TERC*	MYNN	
5q15 (Mitchell et al., 2016)	0.75	1.16	*ELL2*		B & PC differentiation
5q23.2 (Went et al., 2018)	0.43	1.11	*CEP120*		Genomic instability
6p21.3 (Chubb et al., 2013)	0.29	1.21	*PSORS1C2*	POUF51	
6p22.3 (Mitchell et al., 2016)	0.02	1.36	JARID2		
6q21 (Mitchell et al., 2016)	0.21	1.19	*ATG5*	PRDM1	B & PC differentiation
7p15.3 (Broderick et al., 2012)	0.65	1.24	*CDCA7L*		IRF4-MYC
7q22.3 (Went et al., 2018)	0.74	1.12	*CCDC71L*		
7q31.33 (Went et al., 2018)	0.72	1.12	*POT1*	ASB15	Genomic instability
7q36.1 (Mitchell et al., 2016)	0.12	1.22	SMARCD3		Chromatin remodeling
8q24.21 (Mitchell et al., 2016)	0.32	1.15	*CCAT1*	MYC	IRF4-MYC
9p21.3 (Mitchell et al., 2016)	0.63	1.13	*MTAP*		Genomic instability
10p12.1	0.73	1.11	*WAC*		IRF4-MYC
16p11.2 (Went et al., 2018)	0.26	1.15	*PRR14*	SRCAP	IRF4-MYC
16q23.1 (Mitchell et al., 2016)	0.58	1.12	*RFWD3*		
17p11.2 (Chubb et al., 2013)	0.10	1.30	*TNFRSF13B*		B & PC differentiation
19p13.11 (Went et al., 2018)	0.24	1.14	*KLF2*		IRF4-MYC
20q13.13 (Mitchell et al., 2016)	0.08	1.23	*PREX1*		
22q13.1 (Went et al., 2018)	0.66	1.21	*CBX7*		Chromatin remodeling
22q13.1 (Chubb et al., 2013)	0.44	1.22			

*^1^Location of myeloma risk SNP, with the primary paper describing its discovery indicated in square brackets. ^2^ Relative allele frequency using the value reported by Went et al., 2018 which may slightly differ from the original report. ^3^ Odds ratio using the value reported by Went et al., 2018. Carrier status of the risk allele increases the odds of developing myeloma by no less than11% (10p12.1) and no more than 36% (6p22.3). ^4^Gene containing the risk SNP within or outside the gene body, with evidence for genetic interaction affecting gene expression in case of the latter. ^5^Gene target influenced by myeloma risk allele. ^6^ Biological pathway regulated by myeloma risk allele, as proposed by Went et al., 2018. Predictions are based on functional annotation and genetic/biological interference considerations.*

Despite the advances made possible by GWAS, much of the heritable risk of myeloma remains unexplained as of today – a widely known and extensively discussed shortcoming of the method that penetrates the entire cancer field. The 23 loci mentioned above explain but an estimated 16% of the heritability for myeloma in Caucasians, with estimates that a sample size in excess of 5 × 10^4^ is required to explain 80% of the heritability (Went et al., 2018). Some genetic risk variants exhibit myeloma subtype-specific preference, e.g., a variant at *CCND1* is associated with t(11;14)^+^ myeloma (Weinhold et al., 2013), whereas a variant at *CBX7* is linked to subtypes of myeloma that do not carry the translocation (Chubb et al., 2013). This is of interest for working models on tumor development because it points to independent genetic pathways of myelomagenesis.

Four different SNPs – at 3p22.1 (rs1052501), 6p21.33 (rs2285803), 7p15.3 (rs4487645), and 17p11.2 (rs4273077) – were recently identified that independently and significantly increase the risk of MGUS (Weinhold et al., 2014), in ways that, unsurprisingly, overlap with the risk of myeloma (Thomsen et al., 2017). Genetic interaction studies, a newly developed approach to annotate genetic risk patterns with biological functionality, pointed to B cell receptor (BCR) signaling regulated by PREX1 (phosphatidylinositol-3,4,5-trisphosphate dependent Rac exchange factor 1) and SETBP1 (SET binding protein 1) as genetic drivers of neoplastic plasma cell transformation (Chattopadhyay et al., 2018). Genetic interaction analysis like the one used above is able to unify variant pair interaction with genetic networks and pathway enrichment. This holds great promise for closing knowledge gaps on the genetic pathways that govern MGUS and MM as the field moves forward.

### Racial and Ethnic Factors

In the United States, the prevalence of MGUS and frank myeloma is significantly higher in African Americans (AA) than in Caucasian Americans (CA) of European ancestry (Greenberg et al., 2012). For example, a study reporting that the overall myeloma incidence (cases per 100,000 persons) increased from 5.52 in the 5 years period from 1993 to 1997 to 6.08 in the 2008–2012 period (*p* < 0.001) found an increase of ∼13% in CA men (6.39–7.22; *p* < 0.001) but an increase of ∼17% in AA men (13.94–16.15; *p* < 0.01). Thus, in 2012 the myeloma incidence in AA men (16.2 × 10^-5^) was 2.24 times higher than in CA men (7.22 × 10^-5^) and the trend of disparity was increasing (Costa et al., 2017). Another well-established racial difference is the mean age of diagnosing myeloma: it is 4 years younger in AA patients (65.8 years) compared to CA patients (69.8 years) (Waxman et al., 2010). Although confounding effects due to inequalities in health care and a host of environmental and lifestyle factors cannot be excluded, both the higher rate and earlier onset of MM in African Americans support the notion of a racial contribution to the etiology and natural history of MM.

**FIGURE 6:**
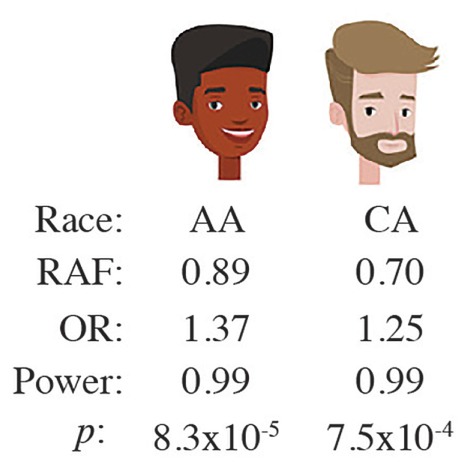
The risk locus at *7p15.3* indicated by SNP, rs4487645, is more strongly associated with myeloma in African Americans than Caucasian Americans.

To determine myeloma susceptibility regions for AA and CA individuals in greater depth, Cozen et al. recently performed a GWAS meta-analysis that included a clever imputation-based fine mapping approach to identifying putative functional variants governing myeloma risk (Rand et al., 2016). The study relied on several loci associated with myeloma risk (Table 1), including variants in *ULK4* (unc-51 like kinase 4); a missense variant in *TNFRSF13B*, which encodes a B cell activating factor (BAFF) receptor from the TNF receptor family called TACI (transmembrane activator and calcium-modulating cyclophilin ligand interactor); SNPs around the promoter and enhancer regions of *CBX7* (chromobox 7); and, importantly, a SNP at *7p15.3* (rs4487645) that was independently confirmed in a GWAS that also implicated the *2q12.3* region in myeloma risk (Erickson et al., 2014). Cozen et al. showed that the *7p15.3* rs4487645 locus exhibits stronger association with MM in AA individuals compared to CA individuals (Rand et al., 2016): 0.89 vs. 0.70 RAF and 1.37 vs. 1.23 OR (at 99% power in both cases and *p*-values of 8.30 × 10^-5^ for AA samples and 7.47 × 10^-4^ for CA samples; Figure 6). To gain insight into the biological function of the *7p15.3* (rs4487645) risk locus in myeloma, Weinhold et al. (2015) carried out an expression quantitative trait locus (eQTL) analysis, which showed that the C risk allele results in elevated *CDCA7L* (cell division cycle associated 7 like) expression compared to the A “non-risk” allele. Following up on that, Li et al. (2016) demonstrated that the C risk allele-dependent increase in *CDCA7L* expression must be attributed to the generation of an IRF4 binding site in the 7p15.3 enhancer. This connected the germline risk of myeloma to a genetic pathway of great significance for myeloma biology: IRF4-MYC. Li et al. also showed that *CDCA7L* mRNA levels may prognosticate survival of patients with myeloma. For example, in the GSE9782 trial, myeloma patients (*n* = 265 total) in the top quartile of *CDCA7L* expression (measured in bone marrow plasma cells) exhibited significantly shorter overall survival than patients in the bottom quartile [*p* = 3.1 × 10^-4^; hazard ratio (HR) = 2.3].

A recent NGS study on tumor-acquired somatic mutations in myeloma reported new insights into racial differences between AA and CA patients (Manojlovic et al., 2017). This included the discovery of significant differences in mutation frequency in 17 genes, with as many as 15 of them (88%) demonstrating a higher mutation frequency in AA than CA myeloma (Table 2, rows 1–15). *IRF4* may be of special interest for two reasons: it is recurrently mutated in CA (3.2%) but not AA patients (Table 2, 2nd to last row) and it is linked to germline risk in the *CDCA7L* locus as described above. What is more, IRF4 is an important transcription factor in the hematopoietic system (Acquaviva et al., 2008) that was identified as a myeloma driver in tumors that carry the IRF4-activating chromosomal t(6;14)(p25;q32) translocation (Iida et al., 1997). IRF4 expression is inversely correlated with clinical outcome of myeloma (Heintel et al., 2008) and IRF4-dependent modulation of Fas-induced apoptosis governs, in part, myeloma survival (Fanzo et al., 2006). Importantly, studies on IRF4 target genes uncovered a positive auto-regulatory loop between *IRF4* and *MYC* (Shaffer et al., 2008). IRF4 is also of interest from the therapeutic angle since it constitutes a “unifying Achilles heel” in myeloma, regardless of the molecular subtypes (Shaffer et al., 2008). The backbone myeloma drug lenalidomide (Len) down regulates IRF4 indirectly because it lies downstream of cereblon (CRBN), the primary target of Len, in the CRBN-IKFZ1/3-IRF4-MYC pathway (Lopez-Girona et al., 2011; Zhu et al., 2011, 2014; Greenberg et al., 2013; Schuster et al., 2014). IRF4K123R is the most common mutant allele in myeloma (Manojlovic et al., 2017; Walker et al., 2015a), with the resulting lysine-to-arginine exchange in the IRF domain of the protein constituting a putative gain-of-function change. Walker et al. (2015a) recently reported that exonic mutations in IRF4, particularly the K123R mutation, result in improved survival in myeloma. This is intriguing as it suggests that evolutionary pressure during tumor progression sometimes selects an allele that restrains tumor aggressiveness instead of facilitating it.

**Table 2:** Genes exhibiting different somatic mutation rates in African American (AA) and Caucasian American (CA) patients with multiple myeloma.

Gene symbol	Gene name	Mutated in AA (%)	Mutated in CA (%)	AA vs CA ratio	*p* value
*ABI3BP*	ABI family member 3 binding protein	3.9	1.0	3.9	0.015
*ANKRD26*	Ankyrin repeat domain 26	3.1	0.2	16	<10^-3^
*AUTS2*	Activator of transcription and developmental regulator	3.9	1.2	3.3	0.028
*BCL7A*	BCL tumor suppressor 7A	3.9	0.8	4.9	0.007
*BRWD3*	Bromodomain and WD repeat domain containing 3	3.9	0.8	4.9	0.007
*DDX17*	DAED-box helicase 17	3.1	0.7	4.4	0.016
*GRM7*	Glutamate metabotropic receptor 7	3.9	1.0	3.9	0.015
*IRF4*	Interferon regulatory factor 4	ND	3.2	N/A	0.041
*MYH13*	Myosin heavy chain 13	3.9	0.8	4.9	0.007
*PARP4*	Poly(ADP-ribose) polymerase family member 4	3.9	1.0	3.9	0.015
*PLD1*	Phospholipase D1	3.1	0.3	10	0.002
*PTCHD3*	Patched domain containing 3	4.7	1.0	4.7	0.003
*RPL10*	Ribosomal protein 10	4.7	1.0	4.7	0.003
*RYR1*	Ryanodine receptor 1	9.4	4.9	1.9	0.045
*SPEF2*	Sperm flagellar 2	3.9	0.8	4.9	0.001
*STXBP4*	Syntaxin binding protein 4	3.1	ND	N/A	<10^-3^
*TP53*	Tumor protein p53	1.6	6.3	0.25	0.035

Myeloma disparity research may be hampered by uncertainty and potential bias introduced by self-reported race rather than objective genetic ancestry data. To address this problem, Rajkumar, Kumar and their associates took advantage of the Precision Medicine Research Array genotyping tool to determine biogeographical ancestry in an unbiased, quantitative manner. Using this method, they were able to demonstrate that a major proportion of the racial AA vs. CA disparity in myeloma is driven by differences in the occurrence of myeloma-associated t(11;14), t(14;16), and t(14;20) translocations (Baughn et al., 2018). A highly promising and practically relevant step toward enhanced understanding of racial disparity in myeloma is the PROMISE study (NCT03689595), which is funded as part of the Stand Up To Cancer Multiple Myeloma Dream Team. The acronym stands for Predicting Progression of Developing Myeloma in a High-Risk Screened Population. The study will enroll an estimated 50,000 participants between 45 and 75 years of age that are either AA individuals (self-identified) or individuals of any race who have a first-degree relative (parent, sibling or child) with frank myeloma or the precursor conditions monoclonal gammopathy of undetermined significance (MGUS) and smoldering multiple myeloma (SMM). The IgM^+^ plasma cell dyscrasia, Waldenström macroglobulinemia, will also be accepted as inclusion criterion. The completion of PROMISE, which is poised to close long-standing knowledge gaps on early stages of myeloma development, is envisioned for 2033. The primary outcome measure is time to progression (TTP) from MGUS/SMM to frank myeloma. The principal goal of the study is the definition of the clinical, (epi)genetic, genomic and/or immune environmental parameters that predict progression to overt cancer. PROMISE is co-led by Drs. Irene M. Ghobrial and Ivan M. Borrello from DFCI and JHSM, respectively. The study will not only address the high burden of myeloma in the African American population but will also catalyze fresh thinking about how to make myeloma a preventable disease.

### Myeloma Risk Alleles Detected in NGS and Immunological Studies

**FIGURE 7:**
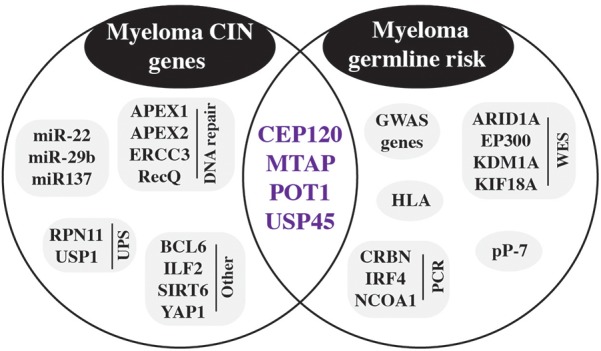
Germline risk contribution to CIN in myeloma. Inherited risk factors detected by GWAS and other methods are shown in the circle on the right. Genes involved in chromosomal instability (CIN) are included in the left circle. Purple genes in the intersection of both circles are germline risk genes that are also involved in CIN.

To date, the strongest molecularly defined risk factor for MGUS and MM is the hyper-phosphorylated paratarg-7 (pP-7) carrier state (Figure 7, right). Paraproteins frequently react with a target protein, named paratarg. Patients with a paratarg-7 (P-7) directed paraprotein carry a hyper-phosphorylated form of P-7 that is inherited in an autosomal dominant manner. The hyper-phosphorylated protein is found in over one third of MGUS/MM patients, with the highest prevalence observed among AA patients. This may explain, in part, the higher frequency of myeloma in the black population. The RR for pP-7 carriers to develop MGUS/MM is 7.9 (Grass et al., 2009). Additional autoantigenic paraprotein targets were subsequently identified, all of which are hyper-phosphorylated in affected patients (Grass et al., 2011). Hyper-phosphorylation may be the result of de-phosphorylation deficiency based on evidence indicating that de-phosphorylation of pP-7 is defective in pP-7 carriers due to inactivation of protein-phosphatase 2A (PP2A) (Preuss et al., 2014). The studies described above and exciting new research by Dhodapkar’s group (Nair et al., 2018) are consistent with the hypothesis that immune responses to post-translationally modified proteins and lipids play a role in myelomagenesis. The association of human leukocyte antigen (HLA) polymorphism with myeloma risk lends further support to this view (Beksac et al., 2016) because HLA proteins are instrumental in initiating T cell-dependent immune responses by virtue of presenting immunogenic peptides to the T cell receptor (TCR). Predisposing or protective associations of HLA polymorphisms with myeloma were identified at the level of individual HLA alleles (A, B, C, DRB3/4/5, DRB1, and DQB1) and the level of haplotype combinations of these loci (Beksac et al., 2016).

PCR-based genotyping methods have provided another approach to discover myeloma risk alleles. One example is the detection of *NCOA1* (nuclear receptor coactivator 1) as a myeloma susceptibility gene in Han Chinese (Peng et al., 2017). NCOA1 is one of three members of the p160/SRC family 33 of proteins and acts as transcriptional coactivator for steroid and nuclear hormone receptors. Another example is variants in *CRBN* (cereblon) and *IRF4* that are involved in myeloma risk and additionally affect therapy responses (Butrym et al., 2016). There is also a growing list of germline variants that influence the survival of patients with myeloma. This includes *BSG* (basigin) and *MCT1* (monocarboxylate transporter 1, officially designated *SLC16A1*) (Lacina et al., 2018), *CDKN2A* (cyclin dependent kinase inhibitor 2A) (Shah et al., 2017), *FOPNL* (FGFR1OP N-terminal like) (Ziv et al., 2015), and *AICDA* (activation induced cytidine deaminase) (Campa et al., 2019).

NGS, particularly WES and exome sequencing in SGS (shared genomic segment) regions, provides yet another approach to uncovering myeloma risk alleles. Examples include *ARID1A* (AT-rich interaction domain 1A) that was detected in a pedigree analysis implicating DNA repair and chromatin remodeling in MM risk (Waller et al., 2018) and *EP300* (E1A binding protein p300) (Bolli et al., 2017), which encodes a histone acetyltransferase (HAT) that regulates transcription via chromatin remodeling. Continuing with the epigenetic theme, WES recently identified in a pedigree of early-onset myeloma the first autosomal-dominant MM predisposition gene: germline N-terminal truncating mutations in *LSD1* (lysine demethylase 1A, official gene symbol *KDM1A*) (Wei et al., 2018). *LSD1* encodes a transcriptional repressor that primarily demethylates histone H3 on lysine 4. The finding that pharmacological inhibition of LSD1 in antigen-challenged mice led to plasma cell expansion and appearance of serum paraproteins supports the contention that the demethylase is involved in malignant plasma cell transformation (Zhu et al., 2011). Last but not least, WES followed by gene burden analysis identified a candidate risk gene, *KIF18A* (kinesin family member 18A, *p* = 3.6 × 10^-6^), that encodes a member of the kinesin superfamily of microtubule-associated molecular motors using hydrolysis of ATP to produce force and movement along microtubules. *KIF18A* displays a distinct pattern of expression across molecular subgroups of MM and is associated with patient survival (Scales et al., 2017). The risk genes mentioned above are included in Figure 7, right.

### Myeloma and Cancer Genetic Susceptibility Syndromes

When faced with the diagnosis of myeloma, patients and relatives will invariably question its cause. As described in greater depth in the two preceding review sections, we now appreciate that a proportion of myeloma is inherited (familial myeloma) and/or facilitated by common low-risk susceptibility alleles (Tables 1, 2). However, clinical oncologists involved in myeloma care should also consider that in some patients with myeloma the proclivity to tumor development may be due to a hereditary cancer syndrome. To date, more than 50 syndromes of this sort are firmly established in clinical practice and the identification of new syndromes is ongoing. A short list of the most relevant conditions, together with affected genes and impact on blood cancers, is presented in Table 3. Syndromes associated with increased risk for liquid and solid neoplasms are shown in the table’s upper half, while syndromes associated primarily with risk for hematopoiectic malignancies are presented in the lower half.

**Table 3:** Hereditary genetic syndromes predisposing, in part, to blood cancers including B-cell lymphoma and multiple myeloma.

Gene symbol	Gene name	Cancer syndrome	Blood cancer (%)
**Group 1^1^**			
ATM	ATM serine/threonine kinase	Ataxia teleangiectasia	30–40
BLM	Bloom syndrome, RecQ like helicase	Bloom syndrome	15
FANCA	FA complementation group A	Fanconi anemia	7–13
MLH1	MutL homolog 1	Constitutional mismatch repair deficiency	33
MSH2	MutS homolog 2		
MSH6	MutS homolog 6		
PMS2	PMS1 homolog 2, mismatch repair system component		
NBN	Nibrin	Nijmegen breakage syndrome	40
NF1	Neurofibromin 1	Neurofibromatosis 1	30–40
PTPN11	Protein tyrosine phosphatase, non-receptor type 11	Noonan syndrome	1
TP53	Tumor protein p53	Li-Fraumeni syndrome	2–4
**Group 2^2^**			
BTK	Bruton tyrosine kinase	X-linked agammaglobulinemia	Unknown
CTLA4^3^	Cytotoxic T-lymphocyte associated protein/antigen 4	Lymphoma predisposition	6
ETV6	ETS variant 6	Familial leukemia	Unknown
FAS	Fas cell surface death receptor	Autoimmune lymphoproliferative syndrome	8–12
FASLG	Fas ligand		
CASP10	Caspase 10		
KLHDC8B	Kelch domain containing 8B	Lymphoma predisposition	Unknown
PAX5	Paired box 5	Leukemia predisposition	Unknown
RBM8A	RNA binding motif protein 8A	Thrombocytopenia absent radius syndrome	1
SBDS	SBDS, ribosome maturation factor	Shwachman-Diamond syndrome	30–40
SH2B3	SH2B adaptor protein 3	Leukemia predisposition	Unknown
SH2D1A	SH2 domain containing 1A	X-linked lymphoproliferative disease	24
WAS	Wiskott-Aldrich syndrome	WAS-related disorders	2–13

*^1^Genes associated with increased risk for liquid and solid neoplasms. ^2^Genes associated primarily with risk for blood cancers. ^3^Newly recognized. For details see: Egg et al., 2018.*

The possibility that cancer may have a hereditary basis was first recognized by Paul Broca (1824–1880) and subsequently confirmed by the discovery of Lynch syndrome (hereditary non-polyposis colon cancer) and Li-Fraumeni syndrome (a.k.a. SBLA or sarcoma, breast, leukemia, and adrenal gland) (Rahman, 2014). Research on retinoblastoma performed by Alfred Knudson (1922–2016) led to the widely known 2-hit model of tumor development, i.e., individuals with hereditary cancer are at increased risk because they carry a dysfunctional germline allele of a gene that normally suppresses cancer formation. Inactivation of the normal copy of that gene on the homologous chromosome dramatically increases the probability to undergo neoplastic transformation. According to this model, cooperative action of a germline mutation (1st hit) and somatic mutation (2nd hit) renders cancer more likely to occur (increased incidence), earlier to occur (during childhood or adolescence), and to occur in multiple tissues and organs (multi-centric tumor development). Knudson’s visionary prediction, first published in 1971 (Knudson, 1971) and confirmed 15 years later with the detection of the first cancer susceptibility and tumor suppressor gene, *RB1* (Friend et al., 1986), inspires myeloma research even today.

Table 3 shows that cancer-predisposing genes that conform to the 2-hit model include *NF1* (neurofibromatosis type 1), *APC* (familial adenomatous polyposis), *BRCA1* and *BRCA2* (hereditary breast and ovarian cancer) and, importantly, p53-encoding *TP53* (Li-Fraumeni syndrome). The latter is particularly relevant for myeloma given the dire prognostic impact of inactivation and mutation of p53 in myeloma (Shah et al., 2018; Walker et al., 2019). Since more than 100 cancer-predisposing genes and their associated syndromes have been identified to date (McGee and Nichols, 2016), it may not be surprising that between 5 and 12% of patients with myeloma are currently believed to harbor at least one cancer-predisposing germline mutation. This emphasizes the importance of obtaining detailed family histories for all patients with myeloma and calls upon physicians to familiarize themselves with inherited pre-disposition syndromes and their presentations. Identifying these syndromes may be a crucial step toward individualized follow-up and treatment of myeloma patients. This should also include the careful evaluation of the genetic fitness of a potential hematopoietic stem cell donor related to the patient.

## Current Research Gaps and Future Directions

### Determining Biological Functions of Myeloma Risk Alleles

The identification of myeloma susceptibility alleles opens the door to fundamental and applied studies on genotype-phenotype correlations, mechanisms of malignant plasma cell transformation, and myeloma progression. Although many questions on how the emerging information on heritable predisposition should be incorporated into the clinical setting remain unanswered, there is no doubt that the new knowledge will eventually lead to more effective cancer treatments, surveillance protocols, and risk-reducing measures. One crucial step to that end is the determination of the biological function of germline myeloma risk alleles. Went et al. recently started this process by assigning some of the myeloma risk loci included in Table 1 to distinct cis-regulatory networks. These were defined with the help of a sophisticated multidimensional genomic analysis that sifted through large ChIP-seq, Hi-C, ENCODE, and eQTL datasets (Went et al., 2018).

Five of the 23 loci listed in Table 1 are involved in the regulation of the IRF4-MYC axis, an important player in myeloma biology. *MYC* is among the most consistently upregulated genes in new myeloma (Zhan et al., 2006) and its overexpression frequently becomes “hard wired” in the course of tumor progression due to illegitimate genetic rearrangements such as complex chromosomal translocations and indels at the *MYC* locus (Bergsagel et al., 1996; Affer et al., 2014). Genes involved in the IRF4-MYC regulatory network are *CDCA7L* at 7p15.3, which has been discussed in section Genetic Pre-disposition to Myeloma above; *WAC* (WW domain containing adaptor with coiled-coil) at 10p12.1, which is believed to be important for RNA processing; *CCAT1* (colon cancer associated transcript 1) at 8q24.21, a long non-coding RNA (lncRNA) with pleiotropic function; *PRR14* (proline rich 14) at 16p23.1, which encodes a protein that supports the structure of nuclear lamina; and *KLF2* (Kruppel like factor 2) at 19p13.11, a zinc finger transcription factor gene that plays key roles in cell differentiation and homeostasis. How these genes interact to regulate the IRF4-MYC axis will be the subject of future studies.

Two additional mechanisms by which myeloma risk alleles included in Table 1 promote oncogenesis concern chromatin remodeling and B cell and plasma cell differentiation. *SMARCD3* (SWI/SNF related, matrix associated, actin dependent regulator of chromatin, subfamily d, member 3) at 7q36.1 and *CBX7* (chromobox 7) at 22q13.1 are thought to be involved in regulation of chromatin remodeling, whereas *ATG5* (autophagy 5) at 6q21, *TNFRSF13B* (TNF receptor superfamily member 13B a.ka. TACI) at 17p11.1 and *ELL2* (elongation factor for RNA polymerase II 2) at 5q15 regulate plasma cell maturation. The risk allele at 5q15 resides in and reduces the activity of a putative gene enhancer, resulting in lower expression of *ELL2* which encodes a key component of the super-elongation complex important for immunoglobulin production in plasma cells (Li N. et al., 2017; Ali et al., 2018).

Inspired by the advances described above, future research will add functionality to the risk loci in Table 1 that have been ignored thus far. *DNMT3A* (DNA methyltransferase 3A) at 2p23.3, which plays a role in myeloma bone disease (Liu et al., 2016), and *JARID2* (jumonji and AT-rich interaction domain containing 2) at 6p22.3, which is important for binding of Polycomb group proteins to target genes, are good starting points because these genes may be considered founding members of a network of predisposition alleles that regulate epigenetics during myelomagenesis. Likewise, much remains to be learned about the role of *PSORS1C2* (psoriasis susceptibility 1 candidate 2) at 6p21.3 and *ULK4* (unc-51 like kinase 4) in the natural history of myeloma. *CCDC71L* (coiled-coil domain containing 71) at 7p15.3 may be involved in plasma cell motility and *PREX1* (phosphatidylinositol-3,4,5-trisphosphate dependent Rac exchange factor 1) at 20q13.13, may play a role in cellular signal transduction. The gap of knowledge regarding the genes and loci mentioned above, and their collaboration as drivers of neoplastic plasma cell development, provides a rich substrate for future investigation.

### Determining Whether Germline Risk Alleles Predispose to Genomic Instability in Myeloma

Three myeloma risk loci in Table 1 harbor candidate genes proposed to regulate genomic stability in tight association with cell cycle progression: *MTAP*, *CEP120*, and *POT1*. *MTAP* (methylthioadenosine phosphorylase) at 9p21.3 plays a major role in polyamine metabolism and is important for the salvage of adenine and methionine. Thus, using methylthioadenosine as substrate, MTAP supplies more than 95% of adenine produced by human lymphoblasts in cell culture. *CEP120* (centrosomal protein 120) at 5q23.2 is required for microtubule assembly, with overexpression of gene product leading to uncontrolled centriole elongation. *POT1* (protection of telomeres 1) at 7q31.33 is part of the shelterin complex important for chromosomal stability (Cacchione et al., 2019). It is possible but has not been demonstrated that another risk locus, *TERC* at 3q26.2, interacts with *POT1* to maintain chromosomal integrity. *TERC* encodes the RNA component of telomerase, which caps eukaryotic chromosomes with repetitive telomere sequences thereby protecting chromosome ends from damage and rearrangement.

7q31.33 is of additional interest because it indirectly involves *ASB15* (ankyrin repeat and SOCS box containing 15) via a looping interaction from an enhancer element (Table 1). ASB15 is a component of the ubiquitin–proteasome system (UPS) that has been implicated in deficient DNA repair in myeloma by another UPS protein: USP 45 (ubiquitin specific peptidase 45). USP45 is a deubiquitylating enzyme (deubiquitylase) that has been recently linked to myeloma risk in high-risk pedigrees (Waller et al., 2018). Two additional enzymes of this type, USB1 and Rpn11, that contribute to maintenance of genomic integrity in myeloma were mentioned above, in section Genetic Pre-disposition to Myeloma of this review. *RFWD3* (ring finger and WD repeat domain 3), a myeloma risk gene at 16q23.1, is also involved in the UPS: it encodes an E3 ubiquitin ligase that participates in DDR in association with the replication protein A complex (Inano et al., 2017). This backdrop suggests that the UPS plays a prominent role in myeloma’s complex regulatory network of predisposition genes that underpin abnormalities in genomic stability and cell cycle progression control. Going forward, elucidating this network in greater depth is an important research objective for myeloma biologists and geneticists.

### Identifying Genetic Modifiers in the Tumor Microenvironment

Myeloma is a complex, interactive system that consists of malignant plasma cells and a large variety of non-malignant bystanders in the bone marrow TME. Bystander crosstalk is likely to modify myeloma development in dependence on genetic predisposition alleles that operate in the TME. However, it is currently unclear what portion of the heritable myeloma risk is determined by the TME because TME risk modifiers remain uncharacterized in myeloma (Flister and Bergom, 2018). To address this shortcoming in future work, it may be useful to define two simple expectations of a hypothetical myeloma TME risk gene. First, it must have a significant association with the disease, similar to the genes in Table 1. Second, it must be expressed and have a biological function in at least one of the TME bystanders mentioned above. Lack of expression in normal or malignant plasma cells would lend additional support to the contention the candidate gene impacts myeloma risk via the TME.

When designing new research on TME risk genes in myeloma, it will be important to keep an open mind and consider pitfalls. For example, it is possible that genetic modifiers of myeloma function in both tumor cells and the TME. Genes with demonstrated functionality in B/plasma cells and at least one bystander cell type may fall into this category. Another possibility is that a given myeloma risk gene functions in the TME under normal conditions but is co-opted by virtue of ectopic expression in tumor cells under aberrant conditions. Be this as it may, additional work is warranted to demonstrate the magnitude with which TME modifiers impact myeloma risk and progression. Assessing the ability of modifiers to drive selection of distinct tumor precursors for distinct TME characteristics will be a difficult but interesting challenge. A recent analysis of The Cancer Genome Atlas (TCGA) data suggests that, in some types of cancer, germline polymorphisms are correlated with somatic mutations (Bailey et al., 2018), however, myeloma was not included. Another open question concerns the significance of TME modifiers for therapy responses and patient outcome. The effect size in myeloma may be larger than in other cancers, considering that BM-secreted factors are well known to modulate the efficacy of chemo- and radiation treatment of cancer.

Envisioned myeloma association studies aimed at differentiating TME and cancer cell autonomous modifiers may find guidance in productive preclinical research projects that relied on animal models of human cancer to identify and validate genetic risk modifiers in the TME. An example from the solid cancer field is research on rat breast cancer susceptibility that led to the discovery of two TME modifier loci. *Mcs5a* influences tumor progression via the immune system (T cells) in a *FBXO10*-dependent manner – analogous to a mechanism in human T lymphocytes (Xu et al., 2014) that is associated with human breast cancer risk (Samuelson et al., 2007). The second modifier locus is linked with *DLL4* and impacts breast cancer growth and metastasis by inducing dysfunctional angiogenesis (Flister et al., 2017). A good example from the blood cancer field that may be highly relevant for myeloma is research at the US National Cancer Institute that uncovered the TME risk gene *Mndal* (myeloid cell nuclear differentiation antigen-like). This gene determines in part the susceptibility of BALB/c mice to inflammation-dependent peritoneal plasmacytoma (Zhang et al., 2009), a first-generation mouse model of plasma cell neoplasia (cf. section Genomic Instability In Myeloma) that was instrumental for elucidating antibody (immunoglobulin) structure and genetics and developing hybridoma technology (Gearhart et al., 2018). The recent demonstration that TME-produced IL-6 is critical for PCT in mice (Rosean et al., 2015) may also be relevant for myeloma because it suggest that human IL-6 promoter polymorphisms associated with predisposition to non-Hodgkin’s lymphoma and Hodgkin’s lymphoma (Peng et al., 2018) are in fact TME risk modifiers of neoplastic B cell and plasma cell development.

### Exploring Potential Links Between the Germline and Somatic Genome in Myeloma

Myeloma subtype-specific associations of germline risk variants and somatic changes in the tumor genome have been reported; e.g., the association of a variant at *CCND1* with t(11;14)^+^ myeloma, or of a variant at *CBX7* with myeloma not harboring chromosomal translocation (see section Racial and Ethnic Factors). However, systematic studies that evaluate the entire somatic and germline genome with the aim of establishing a link between the two are lacking (Geeleher and Huang, 2017). To remedy this shortcoming, it will be necessary to perform genome-wide analyses of germline risk and somatic mutations *within* a large group of patients with myeloma rather than relying on the widely used GWAS approach of the past that compared myeloma patients with normal individuals used as controls. A recent pan-cancer analysis demonstrated the productivity of the new approach (Carter et al., 2017) which may help to bridge the gap between the germline and somatic genome in myeloma. Integrated datasets on germline risk and somatic mutations in myeloma may be key for attacking outstanding questions such as the mechanism that underlies cell type specificity. Why do germline genetic variations increase the propensity of malignant plasma cell development while leaving other blood cell lineages or solid tissues alone? Another important question concerns the extent with which germline genetic variation affects the somatic mutation profile in myeloma and, thereby, influences the Darwinian process of clonal selection and evolution in the course of tumor development.

Another link requiring exploration in future research is the precise contribution of genetic, environmental and lifestyle factors to myeloma. Four specific aims can be readily outlined. First, although it is clear that identifying individuals at increased risk of myeloma allows introduction of appropriate screening and surveillance measures, there is currently an incomplete picture of the absolute risk attributable to inherited, environmental and lifestyle factors. This knowledge gap is not specific for myeloma but true for cancer in general (Simonds et al., 2016). Second, since known Mendelian conditions only account for a very small if not negligible proportion of inherited myeloma risk, and the low-penetrance variants listed in Table 1 only account for an estimated 16% of that risk, it is possible that rare high-penetrance variants segregate in the general population. These should be identified. Third, analogous to cancer at large (Vogtmann and Goedert, 2016), the interaction of the microbiome with heritable risk factors is poorly characterized and requires attention. Fourth, a better understanding of environmental and lifestyle risk factors is desirable. For example, an association of myeloma with diet/obesity has been established (Islami et al., 2018) but the impact of dietary supplements and drugs, e.g., the widely used “baby aspirin” or statin inhibitors (Tsoi et al., 2018), is not known. Elucidating the interplay between these risk factors is no easy task, yet progress promises targeted public health approaches and increased risk factor awareness in the general population. What is more, enhanced understanding of global risk may also facilitate new, stratified interventions tailored to individual patients for precision medicine treatment of MM.

## Author Contributions

All authors wrote the manuscript, designed the tables and figures, and read and approved the final version of the review.

## Conflict of Interest Statement

The authors declare that the research was conducted in the absence of any commercial or financial relationships that could be construed as a potential conflict of interest.
